# A qualitative interview study to determine barriers and facilitators of implementing automated decision support tools for genomic data access

**DOI:** 10.1186/s12910-024-01050-y

**Published:** 2024-05-05

**Authors:** Vasiliki Rahimzadeh, Jinyoung Baek, Jonathan Lawson, Edward S. Dove

**Affiliations:** 1 Center for Medical Ethics and Health Policy, Baylor College of Medicine, 1 Baylor Plaza, Suite 310DF, Houston, TX 77098 USA; 2 Broad Institute of MIT and Harvard, Cambridge, MA USA; 3 School of Law, University of Edinburgh, Edinburgh, UK

**Keywords:** Data access committee, Implementation science, Automation, Decision support, Genomic data, Ethics

## Abstract

Data access committees (DAC) gatekeep access to secured genomic and related health datasets yet are challenged to keep pace with the rising volume and complexity of data generation. Automated decision support (ADS) systems have been shown to support consistency, compliance, and coordination of data access review decisions. However, we lack understanding of how DAC members perceive the value add of ADS, if any, on the quality and effectiveness of their reviews. In this qualitative study, we report findings from 13 semi-structured interviews with DAC members from around the world to identify relevant barriers and facilitators to implementing ADS for genomic data access management. Participants generally supported pilot studies that test ADS performance, for example in cataloging data types, verifying user credentials and tagging datasets for use terms. Concerns related to over-automation, lack of human oversight, low prioritization, and misalignment with institutional missions tempered enthusiasm for ADS among the DAC members we engaged. Tensions for change in institutional settings within which DACs operated was a powerful motivator for why DAC members considered the implementation of ADS into their access workflows, as well as perceptions of the relative advantage of ADS over the status quo. Future research is needed to build the evidence base around the comparative effectiveness and decisional outcomes of institutions that do/not use ADS into their workflows.

**Supplementary Information:**

The online version contains supplementary material available at 10.1186/s12910-024-01050-y.

## Introduction

Genomics is among the most data-prolific scientific fields and is expected to surpass the storage needs and analytic capacities of Twitter, YouTube, and astronomy combined by as soon as 2025 [1]. To meet rising demands for genomic data and their efficient collection and use, national genomics initiatives [2] rely on largescale repositories to pool data resources and incentivize data sharing [3–5]. The “data commons” model has since become the flagship approach for many of these initiatives [6], and prioritizes research collaboration and data access over proprietary exclusion in the data [3]. Data access committees (DACs) are principally charged with ensuring only *bona fide* researchers conducting research permitted by participants’ informed consent are approved to access the data [7]. DACs are typically staffed by research compliance officers, researchers, and sometimes data security professionals. DAC members can be paid or serve as volunteers and, at a basic level, arbitrate access to data given requests meet minimum requirements for data protection and compliance. Critiques of compliance-only responsibilities and the growing appreciation of data privacy risks among the general public has raised questions about whether DACs ought to weigh in on issues of social and scientific value of the data projects [8]. Our prior empirical work [9] suggests there is debate around this scope of DAC oversight, particularly as it relates to considerations of data ethics that are traditionally the domain of institutional ethics committees.

Cheah and Piasecki, for example, propose that DACs have responsibilities to both promote data sharing and protect the interests of individuals and communities about whom the shared data relate: “data access should be granted as long as the data reuse fulfils the criterion of having even a minimal social value, and minimal risk to data subjects and their communities” [7]. In this way, DACs anchor responsible data sharing ecosystems since they govern access to and compliant use of genomic and, increasingly, other health data [10–12].

However, DACs may not contribute to efficient data access provisions as effectively as other review models may allow [13]. In the standard model of data access review, DACs manually review a data requester’s application and assess it against pre-defined criteria. Criteria may include appropriateness of the data requested, data use terms set by data providers, and data privacy and security requirements set by the institution and by law [7]. As with most, if not all, human-mediated activities, manual review of these criteria can be a laborious and error-prone process. For example, DACs may interpret language describing permitted data uses differently, and the terms themselves can sometimes be ambiguous [14]. Faced with this ambiguity, DACs are forced to make subjective judgments about whether requests for data access truly align with permitted data uses, if these permissions have been preserved at all. Inconsistencies in how data use terms are articulated in consent forms and subsequently interpreted and executed by DACs across the biomedical ecosystem [14] can lead to delayed and inconsistent data access decisions, and risk violating the terms by which patients or participants contributed their data in the first place.

Other steps in the data access pipeline can also contribute to research delays. Emerging research suggests there is growing inefficiency, inconsistency, and error in the manual, entirely human-mediated review of data access agreements [13, 15] which are executed in finalizing approved data access requests. Many researchers furthermore still rely on the traditional method of copying-and-downloading data once approved. The copy-download approach multiplies security risks [11], and is quickly becoming unreasonable given the expanding size and complexities of genomic datasets [16, 17].

Standards’ developers and software engineers have therefore sought to semi-automate three axes of data access control within cloud environments – user authentication, review of access requests, and concordance of the proposed research with the data use terms of the data requested [14]. Automated decision support (ADS) systems are a coordinated system of algorithms, software, and ontologies [18] that aid in categorizing, archiving, and/or acting on decision tasks for data access review. The Data Use Oversight System (DUOS) typifies one such automated decision support [19]. In recent beta tests, DUOS was successfully shown to concur 100% of the time with human-decided access requests [15], and also codifies 93% of genomic datasets in NIH’s dbGaP [20].

While ADS can supplement human DACs with semi-automated technical solutions, no systematic investigation has sought to characterize relevant barriers and facilitators to ADS in practice [21]. Moreover, we lack understanding of how DAC members perceive the value added by ADS, if any, on the quality and effectiveness of data access review decisions, as well as what challenges they anticipate in adopting ADS considering the myriad organizational structures within which DACs operate.

Now is an opportune time to study the implementation barriers and facilitators to using ADS solutions for data access as their development converges with large-scale data migration to the cloud that can result in near-instant data access decisions. The genomics community can learn important lessons from previous attempts at (premature) ADS implementation without purposeful stakeholder engagement in public health [22], law enforcement [23] and in clinical care [24]. In this article, we report empirical findings on the “constellation of processes” relevant for implementing ADS for genomic data access management and provide practical recommendations for institutional data stewards that are considering or have already implemented ADS in this context.

## Methods

**Fig. 1:**
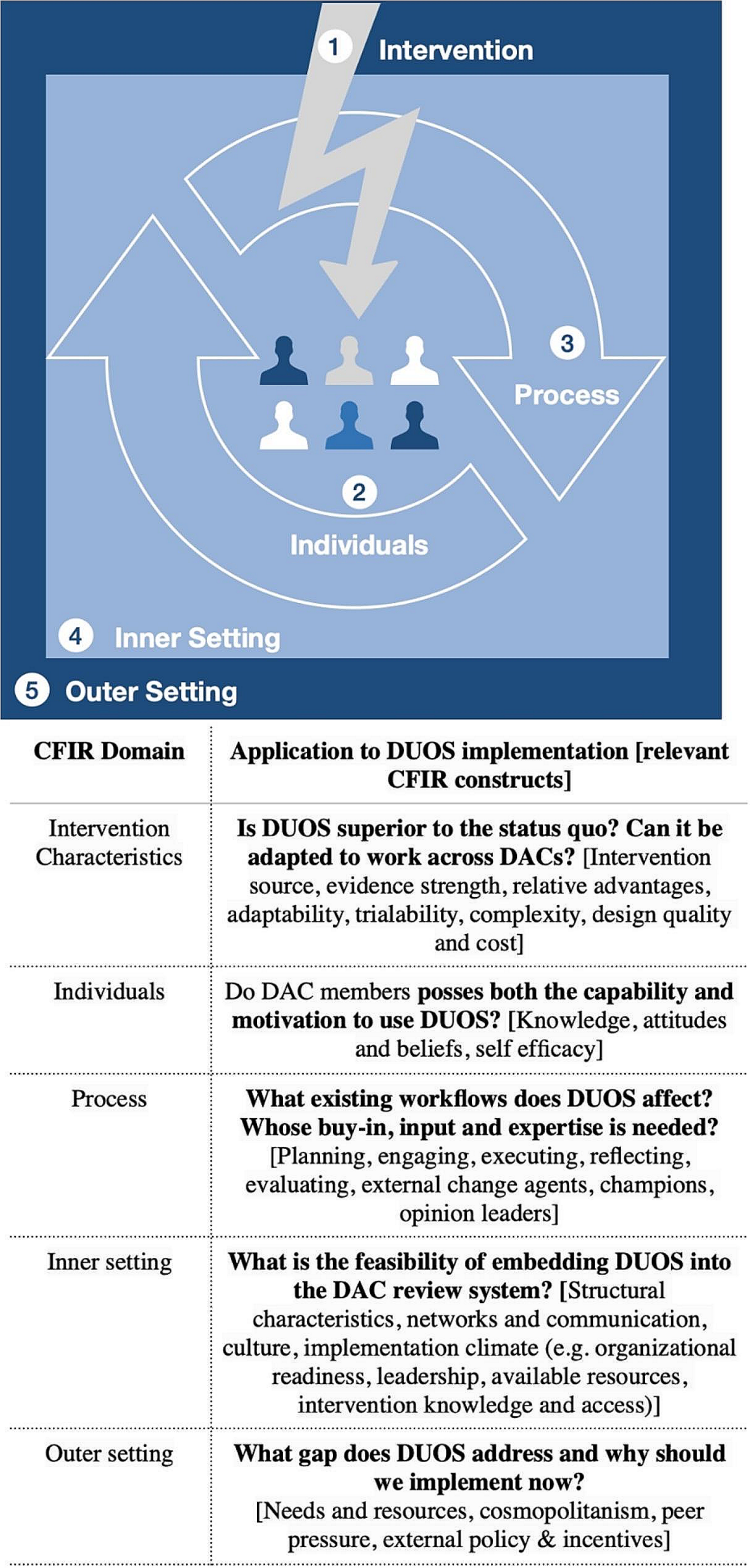
Adapted Consolidated Framework for Implementation Research (CFIR) and associated domains (Intervention Characteristics, Individuals, Process, Inner Setting, Outer Setting) used to structure 13 qualitative interviews on the relevant factors mediating implementation of automated decision support tools for genomic data access management and sharing among publicly funded genomic data repositories worldwide

We conducted a qualitative description study that engaged prospective end users of ADS for genomic data governance to explore: *What are the barriers and opportunities of implementing automated workflows to manage access requests to genomic data collections, and what effect do ADS have on DAC review quality and effectiveness?* We adopted Damshroder and colleagues’ definition of implementation as the “critical gateway between an organizational decision to adopt an intervention and the routine use of that intervention” [25] in order to “study the constellation of processes intended to get an intervention into use within an organization” [25]. We applied the Consolidated Framework for Implementation Research (CFIR) to compare genomic data access processes and procedures to better understand implementation processes for automated workflows to manage genomic data access across international, publicly funded genomic data repositories. The CFIR provides a “menu of constructs” associated with five domains of effective implementation which have been rigorously meta-theorized—that is, synthesized from many implementation theories (Fig. 1). In addition, the CFIR provides a practical guide to systematically assess potential barriers and facilitators ahead of an innovation’s implementation (L. Damschroder et al. 2015). The CFIR is also easily customizable to unveiling bioethical issues during implementation in genomics and has been applied in prior work (Burke and Korngiebel, 2015; Smit et al., 2020).

The interview guide was developed specifically for this study and is available in Supplementary Materials 2.

### Data collection

We conducted a total of 13 semi-structured interviews with 17 DAC members between 27 April and 24 August 2022. Prospective interviewees indicated their interest in being invited to a follow up interview following their participation in a previous survey published elsewhere [9]. All interviews were conducted virtually and audio/video recorded on Zoom. We used validated interview guides from the official CFIR instrument repository (https://cfirguide.org/evaluation-design/qualitative-data/) to probe the barriers and opportunities of implementing ADS solutions for DAC review of data access requests. Interviews lasted between 45 and 60 min and included 29 questions adapted from the CFIR instrument to fit the ADS context (e.g. Inner Setting, Outer Setting, Intervention Characteristics etc.). The specific interview guide used is available in Supplementary Materials 2. Interviewees were also recruited from the Data Access Committee Review Standards Working Group (DACReS WG) chaired by authors VR, JL, and ESD, as well as from an internet search of publicly funded genomic data repositories worldwide.

### Data analysis

We first applied a deductive coding frame to the interview transcripts based on a framework analysis approach (Pope, Ziebland, and Mays 2000) and the publicly accessible CFIR codebook available in the Supplemental Materials 1. To ensure the reliability of conclusions drawn, two independent reviewers (VR and JB) tested the coding schema on three transcripts until reaching a recommended interrater reliability score of 0.83 before analyzing the remaining qualitative dataset. All coding discrepancies during the coding pilot were resolved by consensus discussion.

## Results

- **Geographical, Institutional, and Demographic Background of Participants**

41% of interviewees worked within U.S.-based DACs, while the remaining 59% of interviewees represented DACs at institutions in Canada, the U.K., Spain, Tunisia, Australia, and Japan (Table 1). Nearly 60% of interviewees worked at a non-profit research institute, 24% represented an academic-affiliated research institution, 12% represented a government research agency, and 6% were affiliated with a research consortium. 76% of interview participants identified as female, and 24% as male.

**Table 1:** CFIR Code Application Results

High frequency>=25	Medium frequency10–25	Low frequency< 10
Code (number of code applications)		
Tension for Change (98), Relative Advantage (72), Knowledge &Beliefs about the Innovation (47), Structural Characteristics (36), Planning (33), Cosmopolitanism (32), External Policy & Incentives (30), DAC tools (*code created by the team)* (30), Compatibility (30), Needs & Resources of those Served by the Organization (27), Key Stakeholders (25)	Culture (23), Networks & Communications (22), Relative Priority (21), Cost (21), Adaptability (21), Innovation Source (20), Reflecting & Evaluating (19), External Change Agents (18), Formally Appointed Internal Implementation Leaders (17), Available Resources (16), Individual Identification with Organization (15), Access to Knowledge & Information (14), Evidence Strength & Quality (14), Peer Pressure (12), Other Personal Attributes (12), Opinion Leaders (11)	Individual Stage of Change (9), Goals & Feedback (9), Champions (9), Implementation Climate (8), Engaging (8), Self-efficacy (7), Leadership Engagement (7), Complexity (6), Trialability (6), Learning Climate (6), Characteristics of Individuals (3), Innovation Participants (3), Readiness for Implementation (2), Executing (2), Design Quality & Packaging (1), Process (1)

- **Opportunities for ADS**

We categorized the frequency of CFIR implementation factors referenced in our interviews in Table 2. Our findings suggest that there are three major facilitators to implementing ADS for genomic data governance: (1) external policy and need for efficient workflows, (2) institutional ability to scale the ADS, and (3) interoperability.

**Table 2:** Participant demographics

DAC location	Total % (*n*)
United States	54 (7)
Canada	12 (2)
United Kingdom	6 (1)
Spain	6 (1)
Tunisia	6 (1)
Australia	18 (3)
Japan	12 (2)
**Institutional type**	
Non-profit Research Institute	59 (10)

### External policy and need for efficient workflows

Participants considered adopting ADS to comply with new data sharing mandates from research funders (e.g. National Institutes of Health) and those imposed by peer reviewed journals. The demand for and scope of compliant data access review has had a ripple effect on ethics oversight bodies [26], including DACs, as a result of these new requirements [9]. Most DAC members we engaged with currently perform their reviews manually. Members review all data access requests individually or as a committee and make decisions on each request received in the order they were received. Given the anticipated increase in the number of data access requests [27], our participants noted the reduced workload and costs associated with ADS could contribute to better review efficiencies, without a concomitant loss in review quality and risk of noncompliance with data use conditions.

We found that participants perceived that ADS could reduce DAC member workload by streamlining the intake process for data access requests and verifying that the request matched the terms of use in the original consent obtained at data collection. Indeed, participants noted the initial screening of Data Access Requests (DARs) was a common rate-limiting step in the submission to decision process. DACs often begin the review process by verifying that all necessary information is documented in the request (e.g. study purpose, datasets requested, ethics review). This step can be time-consuming because the requirements can vary depending on the researcher’s institution and the datasets they request. We requested that participants share a copy of their DAR form before, during, or after the interview to compare what information DACs typically required to process a DAR. We found the form fields as well as length of the DAR (from 3 to 18 pages) differed considerably. Our participants believed that this is where ADS could be useful by automatically flagging missing information and documents, verifying the authenticity of a requester’s identity and the submitted documents, and then sending notifications to requesters if more information is needed. As one interviewee put it:

*Because one of the biggest concerns in our DAC is that sometimes it takes too much time to be read by all the nine members. … They’re institutional directors or university professors. So I think it will help. Maybe if you have 50% of the work done by an automated system, so you just have to do the 50%. I think … this will be a good motivation for them saying ‘OK’ [to implement ADS].*

 ‑ Participant M.

### Scalability and cost effectiveness

Participants also believed ADS-enabled workflows could be scalable, cost-effective solutions to management of not just newly generated data, but also for legacy data when grant funding ends because ADS can easily store and quickly present data use conditions and audit past DAC reviews. Two interviewees discussed the challenges of finding cost effective solutions to managing legacy datasets:

Actually there are lots of costs related to data sharing, particularly if I’m sharing data from the 1990s, for example. I don’t have any money or budget anymore to prepare the data [for secondary uses]. … And similarly, when it comes to these reports [on data sharing activities], there’s no extra money for doing the work to create those reports. But we’re having to report back over assets from years, decades in fact. And there was always just a little bit of a hint ‘oh well, maybe we’ll find some money’. No, no, you have to find it out on your own.

 ‑ Participant F.

I mean potentially as we grow over the years, you know what’s going to happen. … we’ve also discussed some scenarios, where, for example, we find ourselves with a larger amount of requests coming in, [and] we only accept applications up to certain days and then, we open this next quarter, close it again. But there potentially could be room for automation depending on the increase in request in the coming years.

 ‑ Participant A.

### Retention and sustainability of human resources

Participants also discussed retention of repository staff and DAC membership as an evolving human resource factor that would motivate ADS adoption. For example, some participants shared that ADS could be helpful when DAC members or data generators leave the institution, disrupting review continuity and consistency. Unlike for large, well-funded government repositories, many DACs at smaller institutions lack human resources to ensure long-term data preservation and access management for data of increasing complexity and volume:

As the program scales, the participant diversity scales, the data diversity scales. I think it is almost impossible to see a scenario where we do not rely on some level of automation to support human decision making about what is responsible use.

 ‑ Participant J.

### Interoperability

According to the DAC members we interviewed, ADS tools could provide centralized, interoperable solutions to facilitate inter-organizational and international data sharing. Participants perceived that ADS could motivate use of standardized request forms, access agreements, dataset identifiers, and methods for verifying researcher identities. For example, one participant commented:

*But this [ADS] will free up a lot of time in the process is it also potentially means that it will become easier for, if you’re working in a team to hand off tasks as well because you will have a single system. … Also, consistency between organizations. If we have multiple organizations take this up, it’s going to mean less lead time. [Let’s] say people take a new job in a new place. We’ll actually have some software that people will recognize and be able to use and uptake, which we’ve been trying to go towards without ethics approval processes within the hospital and health services… [standardized] systems makes it easier for actual communication between organizations on processes, because everyone kind of begins to know what’s happening.*

 ‑ Participant E.

**(b) Barriers to implementing ADS**.

Despite clear advantages of ADS for genomic data access management, our interviewees identified significant barriers to implementation within DAC workflows, including: (1) lower priority compared to more immediate governance challenges, (2) ill equipped personnel and structures within the institution, (3) costs, and (4) degree of human oversight.

### Prioritization

Many participants reported that institutional leadership prioritized other competing research data needs over investing in new data governance structures (e.g. generating quality data, increasing diversity in datasets, collaborating with underrepresented groups of researchers and participants, and releasing datasets). Participants believed researchers in general understand why quality and effective review of data access is important for responsible genomic data sharing but are firstly concerned with data quality. Another suspected reason that ADS implementation ranked lower on institutional priorities was that there had not yet been a significant data incident. As one participant put it:

I don’t think that the program thinks it is a very high priority to streamline any of the [data access oversight] process. I think that it will either take something bad happening and then realizing that we need additional capacities on [DAC], or some other hiccup to really promote that need.

 ‑ Participant O.

Because budgets for data governance are not always included in grants, researchers may be less motivated to invest in the additional, largely unpaid work related to data governance. Insufficient resourcing for data sharing and governance mechanisms prospectively in research study design inevitably challenge the downstream execution of data governance upon deposit of the research data once generated, according to at least one DAC member we interviewed:

We found that some people don’t prioritize [data governance] because it’s not helpful to them, because it’s not our primary function as a department. You know, we’re producing new data. That’s usually what people, researchers are doing. They’re not thinking about what happens to their old data. So, it’s not much of a priority. Having said that, research funders are getting very keen for us to use their data. So, there is that sort of tug [of war]. … If I go into a senior team meeting, you know, something else will be the priority.

 ‑ Participant F.

### Structural characteristics of an organization

We also found a close correlation between several structural characteristics of the institution (e.g. years in operation, number of personnel, and database size) and participants’ perceived barriers to ADS implementation. For instance, many participants served on DACs that were established within the last 1–3 years coinciding with the creation of the institution’s database. As the datasets grow, and more researchers are attracted to the resource, there is greater potential to overwhelm existing management processes. It is precisely at this early juncture that DACs would benefit from weighing their ADS options, and proactively address relevant barriers ahead of any plans for implementation. Some DAC members preferred to gain more experience with existing data access management in these early years of data release before integrating ADS *“because we’re not sure how [name of participant’s country] citizens feel or consider about the automatic decision on data sharing.” Participant K.*

### Cost

While cost was not a primary concern for ADS implementation at well-funded big data repositories, it was a significant barrier for DAC members working at smaller repositories, individual research departments, or research programs associated with a genomics consortium who were more often supported by research grants or contracts rather than an independent funding source.

*“We [data governance office] are supported through project-specific funding. … Governance ends up being a little bit of this indirectly supported component of our work and services. That has limited the ways in which we can innovate around governance. … We don’t have a huge budget.”*

 ‑ Participant N.

Without dedicated budget for human and material resources, some DAC members were concerned that the initial investment in ADS and significant changes to current workflows would be key issues, to say nothing of new education and training materials and updates to internal policies, among other ancillary revisions to internal workflows.

### Lack of human oversight

While some DAC members were enthusiastic about improvements in efficiency and consistency of ADS, participants unanimously rejected the idea of fully automating access management: *“no matter what we do with automation that I feel there always needs to be that human element who’s coming in and checking. So, there will always be that barrier to upscaling” Participant E.* Other participants emphasized that prior to implementation, they would need to gauge how research participants at their own institution as well as the general public would react to ADS for data access review.

Participants were also skeptical that ADS could adequately assess complex, sensitive data reuse issues which they felt required a deep understanding of ethical, legal, and sociocultural contexts within which data were collected, used, and shared. Some DAC members reported asking data requesters to clarify their study purpose and justify their need for specific datasets in recognition of these sociocultural dimensions.

*I’m also someone who thinks that it’s important to be very critical about what’s the nature of the work being done. Maybe it’s solid from a scientific point of view. But are there other concerns from other perspectives that need to be taken into account? That is partly why we have community members on the [committee], and that’s something I’m not sure can be simplified or automated.”*

*However, when it comes to automating anything that requires reviewing information where there might be a lot of nuances, where there might be a lot of interpretation that’s required, I’m a little bit more hesitant simply because I think to some extent you do need some room for a little bit of mulling over the information, … and I think there are some information that come through with requests, that don’t neatly fit into check boxes.*

 ‑ Participant B.

## Discussion

Overall, participants perceived that ADS tools could be well positioned to help DACs streamline data access compliance. While believed to beneficial, ADS solutions were unlikely to immediately or directly advance the research organization’s core mission (e.g. collecting quality data and driving scientific discoveries and innovations). One of the most challenging barriers to implementation is the relative low priority of, and lack of institutional investment in, data infrastructures that could adapt as the dynamics of genomic data generation and storage change over time. Participants tended to regard ADS implementation, as well as data governance workflow solutions, as a lower priority compared to regulatory compliance, investigator support, and database curation, among other competing demands on DAC member time.

Most research grants allow investigators to apply for support for data collection and analysis, but rarely establish actual governance structures needed to stand up access management services. We found that executive buy-in was a major driver for ADS support in the cases of some repositories and the lack or administrative or leadership buy in a major detractor for others, namely repositories at smaller research institutions or laboratories. Therefore, part of the challenge of making ADS adoption a higher institutional priority is convincing institutional leadership of their added value and the net benefit of investing in data governance solutions and infrastructures generally.

Delaying infrastructure upgrades has consequences for the future utility of the repository in the longer term. Some of our study participants, for example, believed researchers were drawn to their databases not because of their data access policies and practices, but because of the quality and diversity of their datasets. However, this quality-driven perspective contrasts with findings from a study of genetic researchers suggesting that ease of access is at least marginally important when choosing a database for their research [28]. We reason that repositories which invest in efficient, scalable, and compliant access decision processes are likely to attract more users to their resources than repositories which do not evolve such processes to meet the pace of data generation and higher data demand. It is also worth noting that funders have a direct role to play in accelerating the pace of data science as researchers are expected to do more with fewer resources and in less time.

Developing more streamlined workflows emerged as a primary benefit that many participants anticipated from adopting ADS. Participants were most enthusiastic about applying ADS for time consuming and tedious tasks, such as preliminary review and quality control checks for data access request forms that are needed to initiate the data access decision process. Applying ADS to facilitate these workflows could free DAC members to dedicate more time to deliberate on more substantive ethics issues raised by data access requests.

While data governance has often been considered auxiliary work, new research findings and new U.S. federal government policies, such as the National Institutes of Health Data Management and Sharing (DMS) Policy, have elevated its importance by placing additional requirements for data sharing [29]. The new DMS Policy was but one example of distinct legislative reforms that have influenced cultures of data sharing shaping DAC work, as well as the institutional practices and governance tools developed to complement this culture. To be sure, such legislative and institutional context influenced participant responses and particular implementation preparedness factors for ADS such as “structural characteristics of the organization.”

The DMS Policy will accelerate the accumulation of an enormous number of datasets. In the absence of interventions, including but not limited to ADS, the DMS Policy will significantly raise costs associated with data storage and management. We concluded from our participants that databases/repositories are frequently developed specifically to share research data generated from federal funds without attention to existing databases and other resources in mind within which to deposit their data. “Blind” database creation is often done with good intentions; however, it can inadvertently introduce myriad access pathways that make the data effectively “shared” but undiscoverable and is another issue where ADS tools could intervene. One participant’s narrative about their need to transfer legacy data from a repository facing permanent closure puts the problem of unsustainable databases in sharp relief. The participant’s example suggested that there is need for more efficient and sustainable solutions for data access management and sharing that can endure even when repositories themselves do not. Moreover, there is reasonable cause to have a contingency plan for publicly funded data shared via non-publicly supported repositories in the event the repository closes or changes in policy or personnel. Standardized ADS solutions could easily interoperate between the two types of repositories and facilitate legacy data transfer, if and when required.

### Limitations

Our results should be considered in light of several methodological limitations. While geographically diverse, many of our interview participants were affiliated with DACs based at large, well-resourced research institutions. It is likely that responses and perceptions of implementation factors related to ADS would differ substantially if more DACs from low- or under-resourced institutions were represented in our sample. Our data collection design relies on self-reports of institutional data access policy and procedures. Many interview participants were aware of the Global Alliance for Genomics and Health, and the data access committee review standards we were principally involved in developing [30]. Thus, while we endeavored to create a safe, open environment for participants to share their honest views, social desirability bias related to our prior work may have influenced how participants responded. Lastly, CFIR predefines sociological constructs relevant to implementation. Our analysis was therefore limited only to those constructs covered in the framework, whereas others might have emerged inductively if we adopted an alternative analytic frame.

## Conclusion

In this article, we reported findings from semi-structured qualitative interviews with DAC members from around the world on the relevant barriers and facilitators of implementing ADS for genomic data access management. Our findings suggest there is general support for pilot studies that test ADS performance for certain tasks in data access management workflows, such as cataloging data types, verifying user credentials, and tagging datasets for use terms. Participants indicated that ADS should supplement, but not replace, DAC member work. This sentiment was especially strong with respect to tasks that were perceived to require sensitivity and human value-judgments such as privacy protections, group harms, and study purpose. Nonetheless, our findings offer cautious optimism regarding the ways in which algorithms, software, and other machine-readable ontologies could streamline aspects of DAC decision-making while also enabling new opportunities for improving consistency and fairness in DAC decisions.

To that end, we conclude with practical recommendations for institutional data stewards that are considering or have already implemented ADS for data access management. First, repositories and institutions that support databases and other resources should prioritize infrastructural upgrades and factor them into associated budgets. Ensuring proper investment in, and human/material resource support for, these upgrades ensures the repository can help ensure its utility even as the complexity and volume of genomic and associated health datasets grow. Second, DACs should prepare to put in place today what data access management and sharing processes they foresee the repository needing tomorrow. For DAC members looking to integrate ADS or other semi-automated tools into their workflows, buy-in from executive leadership should be obtained at the earliest stages of this transition. DAC members should consider substantiating the need for semi/automated solutions with concrete trend data about the frequency of data access requests relative to the time from request to decision and extrapolate these numbers to judge what the anticipated demand for repository will be in 1, 5, and 10 years. Tracking and transparently reporting data access request volume, access decisions, and other committee operations is likewise important not just for internal purposes, but also to demonstrate responsible data stewardship in action to prospective data contributors.

Third, DACs should refrain from implementing ADS wholesale without complementary human oversight of data access request intake and decisions. Pilot testing where ADS tools can be applied to the most time-consuming tasks will require taking inventory of the inputs required for each task along the data access decision workflow. Fourth, DACs should consider what human and material resources will be needed to integrate ADS effectively. These resources include DAC member expertise, computer equipment, and software development, not to mention member education and training resources. Finally, DACs should collaborate on setting standards for how data access requests should be adjudicated and tailor ADS tools in line with these consensus criteria. There is ongoing work to this effect as part of the Ethical Provenance Subgroup of the Global Alliance for Genomics and Health (including the development of an "Ethical Provenance Toolkit"); additional representation from repositories that steward other diverse health datasets would be ideal to coordinate access management strategies across the field.

The explosion in the volume and complexity of genomic and associated health data is converging with the need to manage access more efficiently to these data. Such trends point intuitively to solutions that can help to alleviate, or at least prevent bottlenecks in the access process to preserve the scientific and social value of data generated from public investments in research. To put ADS solutions to the test, future research should compare access decisions and their outcomes between institutions who do/not use such tools for data access management; and examine whether ADS delivers on its efficiency promises and whether it liberates DAC member time previously spent addressing procedural matters – allowing more opportunities for committee deliberation on substantive ethics issues.

### Electronic supplementary material

Below is the link to the electronic supplementary material.

Supplementary Material 1

Supplementary Material 2

## Data Availability

Materials described in the manuscript and data supporting our findings can be made available upon request. All requests should be directed to Vasiliki Rahimzadeh, PhD at vasiliki.rahimzadeh@bcm.edu.
