# Supplementary material for: A qualitative interview study to determine barriers and facilitators of implementing automated decision support tools for genomic data access
Source: BMC Med Ethics. 2024 May 5;25:51. doi: 10.1186/s12910-024-01050-y (PMC11070093; doi:10.1186/s12910-024-01050-y)
Supplement: Supplementary file 2 — Supplementary Material 2 [file 12910_2024_1050_MOESM2_ESM.docx]

**DACReS Interview Guide**

**Part 1 (Introduction)**

1. Welcome and thank the participant for participating in the study.

Good morning/afternoon [name of interview participant]

Thank you for agreeing to talk to me today about your role on your institution’s data access committee. Through our research we are hoping to explore the barriers and facilitators to implementing automated decision tools and other software for reviewing access requests to genomic and related health data. In this study, we are interested in learning about how DAC members review data access requests and the potential barriers and facilitators to implementing the semi-automated system (software) to facilitate data access reviews. The interview builds on the responses we gathered from the survey, which ran earlier in 2022.

| Professional Experience | |
| --- | --- |
| Jinyoung | |
| Background | |
| 1 | Can you tell us how you got involved in your DAC?   - What are the responsibilities you have as a DAC member? |
| 2 | Who are your colleagues? What are their professional background and expertise? |
| DAC operation | |
| 3 | Can you walk me through your review process step by step? |
| 4 | What platforms or tools do you use (for purposes of communication, scheduling, decision-making, data storage/management, etc.)? |
| 5 | Are you and other DAC members satisfied with the current practices? How do people feel about the current review practice/process?   - Could you describe some of the problems (any issues) that have been brought to your attention? |
| Culture/Learning climate (Inner setting) | |
| 6 | To what extent do you feel the DAC embraces new ideas to make improvements to its internal processes? |
| 7 | How would you describe the culture of your organization (e.g., general beliefs, values, assumptions that people embrace)?   - How would this (your organization’s culture) affect how willing it is to embrace new ideas to improve internal review processes? |
| Vaso/Ted | |
| Perceptions of Automated Decision Support system | |
| 8 | How do you define automated decision support tools?   - Can you give examples of any you currently use to support your work? - Do you think automation or implementation of automated decision support tools can be a solution to some of the issues/problems you’ve mentioned? |
| Implementation climate (Inner setting) | |
| 9 | What is the general level of receptivity in your organization to use software and automated decision support tools to support DAC work? |
| Intervention characteristics | |
| [**DUO**](https://github.com/EBISPOT/DUO#what-is-duo) allows to semantically tag datasets with restriction about their usage, making them discoverable automatically based on the authorization level of users, or intended usage. This resource is based on the [OBO Foundry principles](http://www.obofoundry.org/principles/fp-000-summary.html), and developed using the [W3C Web Ontology Language](https://www.w3.org/OWL/). It is used in production by the [European Genome-phenome Archive](https://ega-archive.org) (EGA) at EMBL-EBI as well as the Broad Institute for the [Data Use Oversight System](https://duos.broadinstitute.org/#/home) (DUOS).  [**DUOS**](https://broad-duos.zendesk.com/hc/en-us/articles/360059957092-Frequently-Asked-Questions-FAQs-) enables a researcher to submit a single request for multiple datasets, and the corresponding data access committees to review these requests and provide a response to the researchers. In this process, DUOS also codifies researchers’ requests and datasets’ data use limitations using the Data Use Ontology (DUO) developed by the GA4GH. This makes requests more decipherable both to DACs and DUOS’ matching algorithm, expediting the DAR turnaround time. | |
| Complexity | |
| 10 | Can you explain what you know about DUO/DUOS and how you heard of it?   - How complicated technically would you say DUO/DUOS might be to implement? |
| Tensions for change (Inner setting) | |
| 11 | How essential is DUO/DUOS to meet the needs of DAC members, researchers, and/or data contributors served by your DAC? |
| Stakeholder needs and resources (Inner setting) | |
| 12 | To what extent is the DAC aware of researchers needs and resources and would these guide the decision to implement DUO/DUOS?   - How do you think researchers will respond to DUO/DUOS? - How well do you think DUOS and other automated decision tools would meet the needs of the researchers served by your organization? - Have you elicited information or personal experience from [researchers] to guide your decision whether to implement automated workflow solutions like DUO/DUOS? |
| Evidence strength/quality | |
| 13 | What kind of information or evidence are you aware of, if any, that suggests showing that DUOS will work for your DAC?   - Practice guidelines? Published literature? Co-workers? Other settings? - How does this knowledge affect your perception of DUOS? |
| Compatibility (Inner setting) | |
| 14 | How do you think using DUOS would change/affect your DAC work?   - Would it affect your DAC’s meeting type, communication style, decision-making method, timeline, SOPs, etc.? |
| Jinyoung | |
| Structural characteristics (Inner setting) | |
| 15 | How, if at all, would the specific characteristics of your DAC (e.g., social organization, maturity, size, or physical layout) facilitate/hinder the implementation of DUO/DUOS?   - Are structural changes needed? |
| Relative Advantage | |
| 16 | How would DUOS compare to alternative tools or procedures your DAC may have considered to address workflow challenges in the review process?   - What are the dis/advantages? |
| Adaptability | |
| 17 | Do you think you will need to make any changes to DUOS so that it will work for your DAC?   - Will you be able to make these changes and who decides? |
| Trialability | |
| 18 | Would a pilot help the DAC decide whether or not DUOS could be adopted? |
| Cost | |
| 19 | What are the anticipated costs of implementation and how, if at all, would it affect the decision to implement? |
| Inner setting | |
| Relative priority | |
| 20 | Where does implementing DUO/DUOS rank in importance to other high priority areas related to DAC operations? (high, med, low)   - What other initiatives take priority? |
| Progress evaluation | |
| 21 | Are DAC operations or outcomes currently monitored for success? How would implementation align with the DAC’s goals? |
| Vaso/Ted | |
| Readiness for implementation | |
| Leadership engagement | |
| 22 | What kind of support can you expect from leaders in your organization to help make the implementation successful? |
| Available Resources | |
| 23 | How do you expect to procure sufficient resources, if you don’t already have them to implement and administer DUOS? |
| Access to knowledge & information | |
| 24 | What kind of training, information, and materials about the use of DUOS will be needed? |
| External policies & incentives | |
| 25 | What kind of local, state, or national policies, regulations, or guidelines would influence your decision to use DUO/DUOS? |
| 26 | What kind of financial incentives would influence this decision? |
| Outer setting | |
| Cosmopolitanism | |
| 27 | To what extent do you professionally network with other DACs outside your institution?   - Are they institutions that are similar/dissimilar to yours? Have you discussed DUO/DUOS implementation with them? |
| Peer pressure | |
| 28 | To what extent would implementing DUO/DUOS provide your DAC an advantage over other DACs?   - Are they institutions that are similar/dissimilar to yours? |
| 29 | Would implementing DUOS attract more resources to access your institution's data? |
| Wrap up | |
|  | We’ve come to the end of my questions. Are there other factors or issues regarding implementation that we didn’t cover but which you’d like to share? |
| Thank you | |
